# Hydrogenative-PHIP polarized metabolites for biological studies

**DOI:** 10.1007/s10334-020-00904-x

**Published:** 2021-02-02

**Authors:** Francesca Reineri, Eleonora Cavallari, Carla Carrera, Silvio Aime

**Affiliations:** 1 Department of Molecular Biotechnology and Health Sciences, University of Torino, Via Nizza 52, Turin, Italy; 2 Institute of Biostructures and Bioimaging, National Research Council, Via Nizza 52, Turin, Italy

**Keywords:** Hyperpolarization, Metabolism, Para-hydrogen, NMR

## Abstract

ParaHydrogen induced polarization (PHIP) is an efficient and cost-effective hyperpolarization method, but its application to biological investigations has been hampered, so far, due to chemical challenges. PHIP is obtained by means of the addition of hydrogen, enriched in the para-spin isomer, to an unsaturated substrate. Both hydrogen atoms must be transferred to the same substrate, in a pairwise manner, by a suitable hydrogenation catalyst; therefore, a de-hydrogenated precursor of the target molecule is necessary. This has strongly limited the number of parahydrogen polarized substrates. The non-hydrogenative approach brilliantly circumvents this central issue, but has not been translated to in-vivo yet. Recent advancements in hydrogenative PHIP (h-PHIP) considerably widened the possibility to hyperpolarize metabolites and, in this review, we will focus on substrates that have been obtained by means of this method and used in vivo. Attention will also be paid to the requirements that must be met and on the issues that have still to be tackled to obtain further improvements and to push PHIP substrates in biological applications.

## Introduction

Hyperpolarization techniques allow to increase the sensitivity of Nuclear Magnetic Resonance (NMR) based techniques (MRS and MRI) by orders of magnitude [1, 2] and their application to biological systems opened new perspectives in the field of diagnostics [3, 4].

ParaHydrogen induced polarization (PHIP) was the first in vivo translated hyperpolarization technology and, in these first studies, hyperpolarized molecules were used as angiographic tracers [5, 6] outlining the advantage that the ^13^C images of the injected hyperpolarized probes can be obtained within few tens of seconds after injection.

Soon after that, thanks to the set-up of dissolution-dynamic nuclear polarization (d-DNP) [2, 7], it was clear that the development of hyperpolarization techniques offer the possibility of gaining information about the real-time in vivo kinetics of the metabolic transformations [8] involving injected hyperpolarized substrates. Metabolic studies enabled by d-DNP become possible, in the clinics, due to significant investments by the stakeholders in this technology.

While, in principle, d-DNP can be applied to any substrate, some requirements must be met to allow hyperpolarization such as the formation of a glassy solid solution of the substrate and the free electron radical. Although significant technological advancements have been made to avoid the consumption of cryogenic fluids [9] and to obtain solutions of HP products ready for in-human use [10], the high costs and the complexity associated with d-DNP technology, combined with relatively low production rate, may represent a limitation to the adoption of this methodology.

Approaches exploiting ParaHydrogen induced polarization (PHIP) [11–15] could be attractive alternatives because of their markedly lower costs and instrumentation demands associated to much higher hyperpolarization rates (minutes to seconds, that, in some cases, allow a continuous agent production) [16–18].

**Fig. 1:**
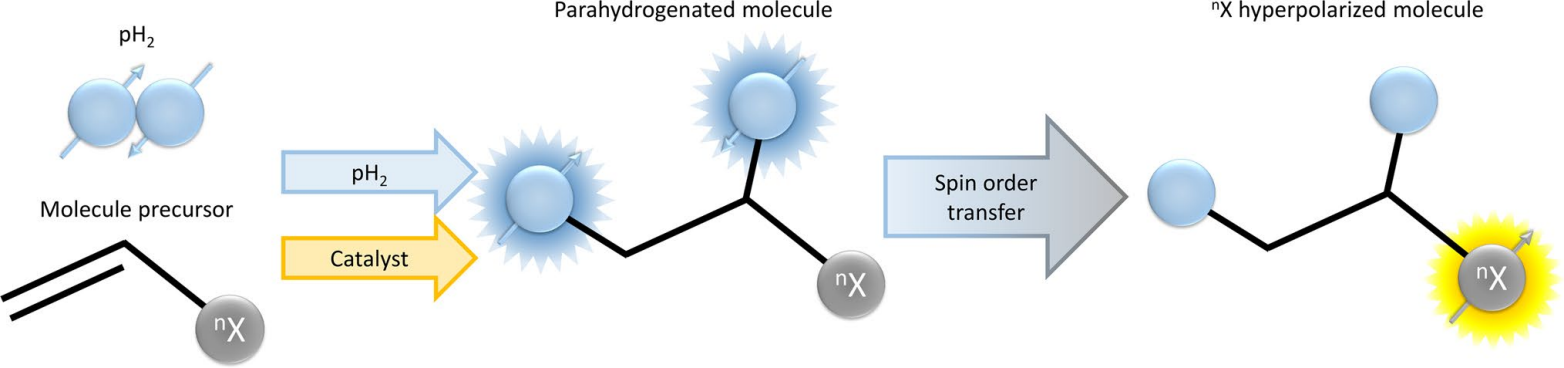
Scheme representing hydrogenative-PHIP (h-PHIP). ^n^X is the hyperpolarized heteroatom (usually a ^13^C spin). In the first step, the unsaturated precursor of the target product is hydrogenated, using parahydrogen and an hydrogenation catalyst. Then, parahydrogen spin order is transformed into heteronuclear polarization by means of spin order transfer (SOT). In this case, the heteroatom is situated only two and three bonds away from the parahydrogen protons

Hydrogenative PHIP (h-PHIP) results from the addition of hydrogen, enriched in the para-isomer, to unsaturated substrates, catalyzed by organometallic complexes (Fig. 1). Therefore, h-PHIP polarized molecules contain two protons, that derive from the pairwise addition of hydrogen to the unsaturated precursor. This clearly represents a strong limitation to the PHIP polarizable substrates. In this context, the introduction of non-hydrogenative PHIP, named Signal Amplification by Reversible Exchange (SABRE), brilliantly circumvented this central issue [19]. This hyperpolarization method is based on the reversible formation of a ternary adduct between the target molecule, parahydrogen and an Iridium complex [20]. The hyperpolarization level gained on the products of SABRE depends on the exchange kinetics of the adduct and is a function of several experimental conditions, such as hydrogen pressure, use of co-solvents and strength of the magnetic field at which the process is carried out [21, 22]. More recent studies about pyruvate hyperpolarization using SABRE [23] are paving the way to the in vivo application of this powerful and versatile technique [24].

Another significant feature of PHIP polarized materials is given by the attainment of hyperpolarized coherences on protons, that can be detected using dedicated pulse sequences such as the OPSY [25] introduced by Duckett et al. Acquisition of hyperpolarized proton signals would be a huge advantage for MRI applications and, in particular, for translation to the clinics, because heteronuclear coils are not usually implemented on clinical MRI scanners. Nevertheless the relaxation rate of ^1^H signals remains a major issue, at least in conditions that are compatible with in-vivo applications (i.e., aqueous solutions of biocompatible molecules, containing oxygen). Locking these hyperpolarized signals into singlet states [26–28] can be a promising way to overcome this hurdle [29, 30].

Many strategies have been introduced, in this research field, to improve the hyperpolarization efficiency, to widen the pool of available substrates and to tackle the more general issue of the limited lifetime of hyperpolarization. This review does not aim to provide an overview of all of them, for which other works have been published recently [15, 31], but it will be mainly focused on h-PHIP.

Nevertheless, the molecules that have been hyperpolarized by means of PHIP, with a biological application in mind, will be reported in the first part of the review. Only few of them have been used for investigations in cell cultures and in vivo*.* Attention will be focused on those molecules and on the methods that made possible the biological application.

Some major issues have still to be solved, and namely the hyperpolarization level, the concentration of the substrates and bio-compatibility of the hyperpolarized solutions. The routes that can be pursued, to tackle these main hurdles and push PHIP substrates in biological studies will be described.

## PHIP polarized molecules for biological studies

During two decades, many biologically significant molecules, some of which are metabolites, their mimics, nutrients and drugs, have been hyperpolarized by means of parahydrogen based methods. Many of these substrates have been reported in Table 1, together with some of the features that are relevant for the biological studies, which are: (i) the solvent in which the final HP product is obtained; (ii) the concentration of the HP agent; (iii) the hyperpolarization level on protons and/or on carbon signals.

**Table 1:** PHIP polarized substrates for biological purposes. Some of these molecules have been hyperpolarized using different PHIP based methods. In these cases, they are listed in chronological order

Molecule	pH_2_	Solvent	[HPmolecule]	Catalyst and HP method	^1^H HP	^13^C HP	References
Metabolites							
Acetate	92%	D_2_O	75 mM	Rh PHIP-SAH		Yes, n.d	[32]
1-^13^C-acetate	–	CD_2_Cl_2_	50 mM	Ir SABRE-RELAY	–	48 fold@ 9.4 T	[33]
	80%	Methanol-d_4_/ethanol-d_6_	30-40 mM	Ir SABRE	–	108 fold@ 9.4 T	[34]
	92%	Methanol-d4/D2O	0.9 mM	Rh PHIP-SAH		19.3%	[35]
Acetate-d_3_ 1-^13^C-acetate-d_3_	92%	Acetone-d_6_/D_2_O or Methanol-d_4_/D_2_O	0.5 mM	Rh PHIP-SAH		3.6%	[36]
1-^13^C-fumarate	86%	D_2_O	45 mM	Ru (water soluble) h-PHIP	–	24% @ 14.1 T	[37]
1-^13^C-lactate	92%	H_2_O	40–60 mM	Rh PHIP-SAH	Yes	2.9% @ 14.1 T	[38]
1-^13^C-phospholactate-d_2_	90%	D_2_O	25 mM	Rh (water soluble) h-PHIP	–	15% @ 47.5 mT	[39] [40]
1-^13^C-pyruvate	92%	H_2_O	30–40 mM	Rh PHIP-SAH	n.d	3.5% @ 14.1 T	[41, 41]
1-^13^C-pyruvate	92%	Acetone-d_6_/Methanol-d_4_/D_2_O	0.75 mM	Rh PHIP-SAH		3.4%	[36]
1-^13^C-pyruvate 2-^13^C-pyruvate	93%	Methanol-d_4_	25-30 mM	Ir SABRE	–	1% @ 9.4 T 0.6% @ 9.4 T	[23]
1-^13^C-pyruvate	–	CD_2_Cl_2_	50 mM	Ir SABRE-RELAY	–	0.039%* @ 9.4 T (48 fold)	[33]
1,2-^13^C_2_-pyruvate	99%	Methanol-d_4_	30 mM	Ir SABRE	Yes	1.7% @ 9.4 T (C1 and C2 average)	[43]
1,2-^13^C_2_-pyruvate	93%	Methanol-d_4_	25-30 mM	Ir SABRE	–	1.8% (C1) 1.6% (C2)@ 9.4 T	[23]
1,2-^13^C_2_-pyruvate	93%	D_2_O/Ethanol-d_6_ (70:30)	0.4 mM	Ir SABRE	–	n.d.	[23]
1-^13^C-succinate-d_2_	90%	D_2_O	30 mM	Rh (water soluble) h-PHIP	–	28% @ 47.5 mT	[39]
1-^13^C-succinate-d_2_	90%	H_2_O	5 mM	Rh (water soluble) h-PHIP		11%	[44]
Urea-^13^C	–	CD_2_Cl_2_	50 mM	Ir SABRE-RELAY	–	0.15%* @ 9.4 T (182 fold)	[33]
Urea-^13^C-^15^N_2_	–	CD_2_Cl_2_	25 mM	Ir SABRE-RELAY	–	0.33%* on ^13^C @ 9.4 T (408 fold) ^15^ N HP yes, n.d	[33]
Amino acids and their derivatives							
1-^13^C-glycine	80%	D_2_O	0.6 mM	NAC@Rh h-PHIP	1.14%	0.29% @ 7 T, 353 K	[45]
1-^13^C-alanine	80%	D_2_O	0.6 mM	NAC@Rh h-PHIP	1.02%	0.25% @ 7 T, 353 K	[45]
*N-*acetyl-AAs (Phenylalanine)	50%	Methanol-d_4_	40 mM	Rh h-PHIP	Yes	1.3% @ 11.7 T, 298 K of fully deuterated *N*-acetyl-1-^13^C-phenylalanine	[46]
*N*-unprotected AA derivatives (esters)	95%	D_2_O	2 mM approx. (5–10% of 25 mM)	Rh (water soluble) h-PHIP	0.7% Gly and Ala deriv 0.5% Gln @ 14.1 T, 353 K	4.4% alanine deriv. @ 9.4 T	[47]
Nitrogenous bases							
Glycine-histidine-phenylalanine	50%	Methanol-d_4_	~ 2 mM	Ir SABRE	Yes, n.d	–	[48]
Adenine Adenosine	95%	Methanol-d_4_	10 mM	Ir SABRE	Yes, n.d. 0.4% @ 9.4 T	–	[16]
Adenine	80–90%	Methanol-d_4_	n.d	Ir SABRE-SHEAT	–	0.06%*** ^15^ N @ 8.5 T	[49]
Adenosine 5′-triphosphate (ATP)	–	CD_2_Cl_2_	50 mM	Ir SABRE-RELAY	–	0.063%*, 0.042%*, 0.026%* on ^31^P @ 9.4 T (48,32,20 fold)	[33]
Purine		Methanol-d_4_	100 mM	Ir SABRE	Yes, n.d		[19]
Nutrients							
d-glucose	–	Acetone/ DMSO	50 mM	Ir SABRE-RELAY	0.13%* @ 9.4 T (40 fold)	–	[33]
d-GLUCOSE-^13^C_6_	–	CD_2_Cl_2_	50 mM	Ir SABRE-RELAY	–	n.d	[33]
d-glucose-^13^C_6_ Fructose-^13^C_6_-d_7_	99%	CD_2_Cl_2_/DMF	20 mM	Ir SABRE-RELAY	–	1.1% ^13^C @ 1 T 0.2% ^13^C @ 9.4 T	[50]
Nicotinic acid (vitamin B_3_)	–	Methanol-d_4_	40 mM	Ir SABRE	2.6%*/total ^1^H signal @9.4 T (800 fold)	–	[51]
Nicotinamide	50%	Methanol-d_4_	n.d	Ir SABRE-SHEAT	–	3.4% ^15^ N @ 8.5 T	[49]
Nicotinamide-d_2_	–	Methanol-d_4_ Ethanol-d_6_	20 mM	Ir SABRE	4.1%** 2.1%**	–	[21]
Drugs							
Alectinib (potential chemoterapeutic)	80–90%	Methanol-d_4_	n.d	Ir SABRE-SHEAT	–	1.5% on ^15^ N @ 8.5 T	[49]
CHCA (chemoterapeutic)	80–90%	Methanol-d_4_	n.d	Ir SABRE-SHEAT	–	1.1% on ^15^ N @ 8.5 T	[49]
Isoniazid (antitubercolosis)	92.5%	Methanol-d_4_	4 mM	Ir SABRE	8% @ 16.4 T, 319 K	–	[52]
Metronidazole (antibiotic)	75–85%	Methanol-d_4_	20 mM	Ir SABRE-SHEAT	–	34% on ^15^ N @ 9.4 T, 300 K	[49, 53]
PET tracers *O*-(2-[^18^F]fluoroethyl)-l-tyrosine ([^18^F]FET) [^18^F]fallypride ([^18^F]FP)	92%	Acetone-d_6_/D_2_O and ethanol-d_6_/D_2_O MeOD-d4	12 mM (12% of 100 mM) 8 mM	Rh h-PHIP	4.5%*@ 7 T 3.5%* @ 7 T, 333 K	4–7.4% @ 7 T, 333 K 3.1% @ 7 T, 333 K	[54]
Pyrazinamide (antitubercolosis)	92.5%	Methanol-d_4_	4 mM	Ir SABRE	8% @ 16.4 T, 319 K	–	[52]

*To facilitate comparison between the polarizations reported in this table, the P% was calculated as the ratio of the reported signal enhancement with respect to the maximum signal enhancement at the experimental used magnetic field and temperature

**Maximum polarization on one of the bis-deuterated analogs

***Enhancement averaged over all ^15^N sites

Among those, attention must be paid to that an aqueous solution, suitable for in vivo administration through intra-venous bolus injection, is obtained. In general, in the development of a hyperpolarized probe, the toxicity of the substrate and the *T*_1_ relaxation of the HP signal play a central role. When PHIP polarization is considered, the solution in which the HP product is obtained becomes a central issue and carrying out the hyperpolarization step in an organic solvent becomes, often, unpractical. Unfortunately, the catalysts usually employed for the parahydrogen hyperpolarization are more active in organic solvents and most of the highest hyperpolarization levels [21] have been obtained using methanol or acetone (see Table 1).

The requirements about the purity and the sterility [10] of the solutions become much stringent if in-human applications are pursued. Albeit the clinical translation does not seem close, at present, for PHIP polarized substrates, these aspects must be kept in mind during the development of these agents.

Nevertheless, in vivo pre-clinical studies are possible provided that physiologically compatible solutions are obtained, at physiological pH (6.5–7.4) and osmolarity in the range of 250–500 mOsm/L (physiological osmolarity 290 mOsm/L). Cytotoxicity studies can also be carried out to assess the biocompatibility of the substances. These requirements have been met for some of the PHIP polarized substrates, as will be detailed in a following section. In the end, it can also be reminded that the first in-vivo studies employing PHIP polarized substrates made use of a toxic molecule [5, 6] which was employed to obtain angiographic ^13^C images.

The high amount of the agents used for in-vivo experiments must also be considered, in fact, the concentration of HP pyruvate used for pre-clinical studies is a few tens of millimolar (80 mM) and ranges from 80 to 250 mM [55, 56] in humans. The biocompatibility of the substrate can become an issue, at this dose.

Furthermore, the hyperpolarization carried out by means of PHIP must be able to provide doses of the HP agent at those concentrations (or higher) in few seconds, to minimize hyperpolarization decay due to relaxation.

Considering all these features, i.e., the biocompatibility of the solution of the HP agent, the high concentration and the polarization level of the probe, a few of the PHIP polarized substrates that are listed in Table 1 become closer to the biological applications than other. These substrates, together with the h-PHIP based methods used for their hyperpolarization, will be described in more details in the following paragraphs.

## h-PHIP polarized metabolites

### [1-^13^C]pyruvate, [1-^13^C]lactate and [1-^13^C]acetate

Among all the hyperpolarized bio-molecules, [1-^13^C]pyruvate is, by far, the most widely investigated metabolite. Pyruvate is at the intersection of pathways important for glucose and energy homeostasis. Thus the information that may be gained about pyruvate metabolism is crucial in tumours’ characterization as well other pathologies. The translational path of d-DNP methodology to the clinics has yielded very promising results on advanced trials with [1-^13^C]pyruvate metabolism in prostate [57] and breast cancer [56]. Moreover, important studies dealing with the characterization of energy metabolism in human brain tumors [58] and in the investigation of heart metabolism [59] have been published.

Hyperpolarized [1-^13^C]lactate [59, 60] and [1-^13^C]acetate [61] have also been the subject of in vivo metabolic investigations in rodents (mice or rats), using d-DNP polarized substrates.

Since de-hydrogenated precursors of these molecules were not available, their hyperpolarization by direct incorporation of para-enriched hydrogen was precluded and the d-DNP route was considered as the only viable possibility.

**Fig. 2:**
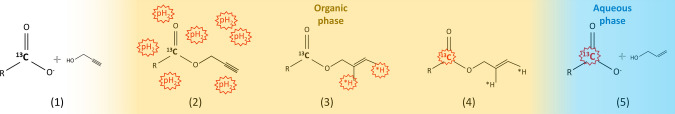
Scheme reporting the key steps of the PHIP-SAH procedure: (1) functionalization of the target molecule with an unsaturated alcoholic group (propargyl or vinyl alcohol); (2) and (3) hydrogenation using parahydrogen of the unsaturated moiety; (4) polarization transfer to the ^13^C carboxylate spin by means of magnetic field cycle (MFC); (5) hydrolysis and phase transfer of the sodium salt from the organic to the aqueous phase

The Side Arm Hydrogenation (SAH) strategy (Fig. 2) overcomes this issue by esterification of the carboxylate with an unsaturated alcohol (the side-arm) [32].

The unsaturated moiety in the added ester functionality is hydrogenated with parahydrogen in an organic, hydrophobic solvent, i.e., chloroform, to pursue an efficient hydrogenation. Then one proceeds with the hydrolysis of the ester and a phase separation step of the now hyperpolarized sodium salt of the carboxylate containing compound.

Parahydrogen spin order is transferred to ^13^C spin polarization through small J-couplings (< 1 Hz). In the reported works, this has been achieved by means of the non-selective polarization transfer procedure named magnetic field cycle (MFC) [62]. Alternative methods for the transformation of parahydrogen spin order into heteronuclear magnetization, through the application of RF pulses, have also been proposed and will be described later.

The hydrolysis reaction is carried out by injecting a aqueous heated solution of sodium hydroxide into the organic solvent containing the hydrogenated ester and the hydrogenation catalyst to obtain a two phase system, where the aqueous phase contains the hyperpolarized sodium salt. By this procedure, almost 99% of the metal containing catalyst remains in the organic phase [31].

In principle, this procedure can be applied to any molecule containing a carboxylic group and it was used to obtain aqueous solutions of ^13^C hyperpolarized [1-^13^C]pyruvate, [1-^13^C]acetate [32, 32] and l-[1-^13^C]lactate [64], suitable for biological applications. The in vitro transformation of hyperpolarized l-[1-^13^C]lactate into [1-^13^C]pyruvate has been tested in a buffered solution of lactate dehydrogenase (LDH), coenzymes and sodium pyruvate and in a suspension of breast cancer (TS/A) lysed cells.

**Fig. 3:**
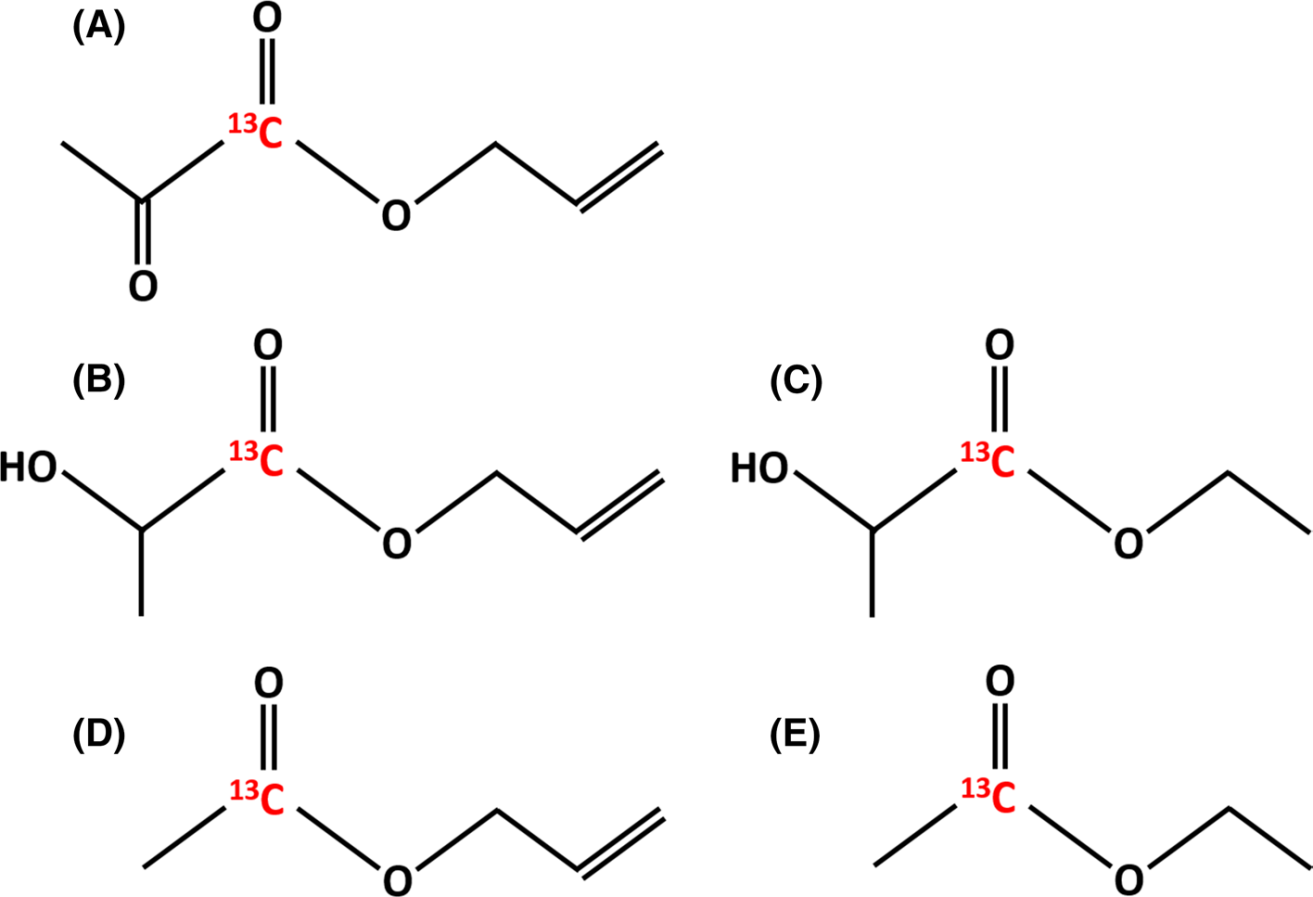
Chemical structures of compounds that have been used in the PHIP-SAH procedure. **a** [1-^13^C]allyl pyruvate, **b** [1-^13^C]allyl lactate, **c** [1-^13^C]Ethyl Lactate, **d** [1-^13^C]allyl acetate, **e** [1-^13^C]ethyl acetate

Propargyl esters are commonly used in this process, but esters from other unsaturated alcohols have been tested (Fig. 3). For instance, vinyl and allyl esters have also been successfully tested [63, 64] (Salnikov et al. 2019). However, the vinyl ester lactate derivative has led to a lower ^13^C polarization than the propargylic one, under the same hydrogenation and polarization transfer condition, as reported in Table 2 [64]. This is probably due to the less efficient hydrogenation of the double bond.

**Table 2:** PHIP-SAH polarized derivatives: ^13^C polarization is reported together with the experimental conditions (solvents, catalyst, parahydrogen percentage) in which this polarization has been obtained

HP agent	pH_2_	Solvent	Catalyst	Measured polarization % (ester)	Measured polarization % after cleavage	References
[1-^13^C]allyl pyruvate	86%	CDCl_3_ +Ethanol	[Rh(COD)(dppb)]BF_4_	6.2 ± 0.3	3.5 ± 0.5	[111]
[1-^13^C]allyl pyruvate	85%	CD_3_OD	[Rh(nbd)(dppb)]BF_4_	5.4		[134]
[1-^13^C]allyl pyruvate	85%	D_2_O	[Rh-(nbd)(Ph((CH_2_)_3_SO_3_-)P-(CH_2_)_4_-PPh((CH_2_)_3_SO_3_-))]-BF_4_	0.82		[134]
[1-^13^C]ethyl pyruvate	85%	CD_3_OD	[Rh(nbd)(dppb)]BF_4_	0.88		[134]
[1-^13^C]propyl pyruvate	85%	CD_3_OD	[Rh(nbd)(dppb)]BF_4_	0.49		[134]
[1-^13^C]allyl lactate	86%	CDCl_3_	[Rh(COD) (dppb)]*BF*_*4*_	4.5 ± 0.5	2.9 ± 0.2	[64]
[1-^13^C]ethyl lactate	86%	CDCl_3_	[Rh(COD) (dppb)]*BF*_*4*_	2.0 ± 0.1		[64]
[1-^13^C]allyl acetate	50%	CDCl_3_	[Rh(COD) (dppb)]*BF*_*4*_	2.3		[62]
[1-^13^C]ethyl acetate	85%	CD_3_OD	[Rh(COD) (dppb)]*BF*_*4*_	4.4		[134]
[1-^13^C]ethyl acetate	85%	D_2_O	[Rh-(nbd)(Ph((CH_2_)_3_SO_3_-)P-(CH_2_)_4_-PPh((CH_2_)_3_SO_3_-))]-BF_4_	2.1		[134]
[1-^13^C]propyl acetate	85%	CD_3_OD	[Rh(nbd)(dppb)]BF_4_	0.35		[134]

The hyperpolarized ester of pyruvate, lactate and acetate created and studied so far using the PHIP-SAH procedure are presented in Table 2.

### Metabolic studies and Bio-compatibility of PHIP-SAH hyperpolarized pyruvate

**Fig. 4:**
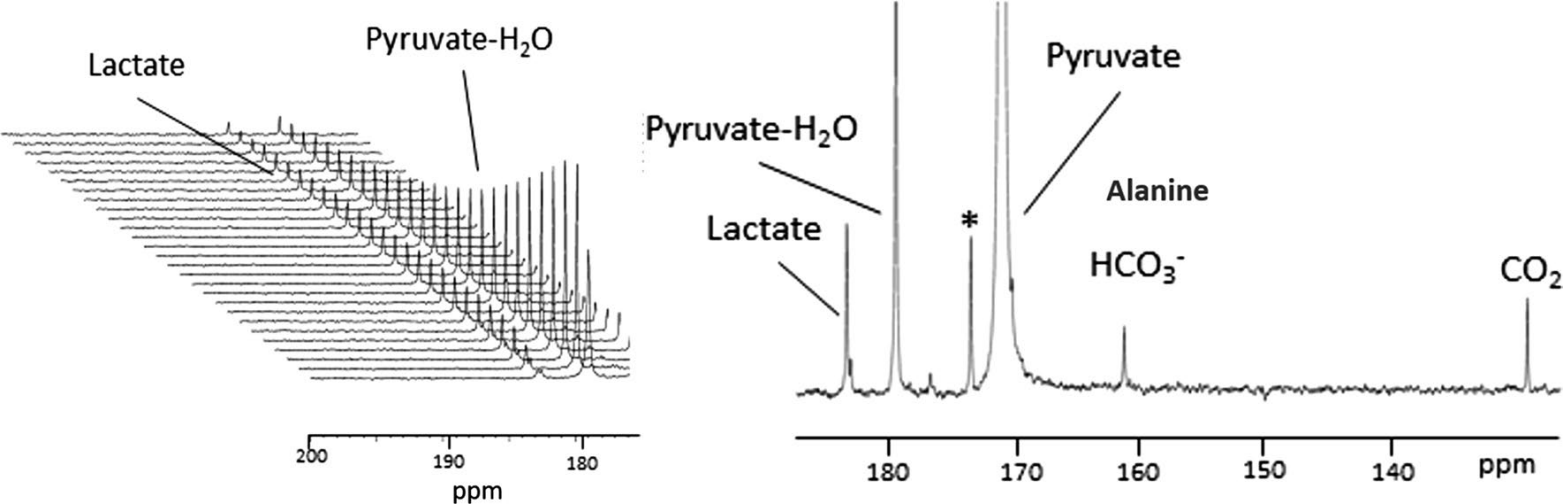
(Left) Stacked plot of the successively acquired ^13^C NMR spectra, following to the injection of HP [1-^13^C]pyruvate through a 168FARN cells suspension; right) one of the ^13^C NMR spectra of the series [65]

Recently the first in vitro applications of PHIP-SAH hyperpolarized [1-^13^C]Pyruvate have been reported for assessing upregulated glycolysis in murine breast cancer cell lines (168FARN and 4T1) (Fig. 4) [65] as well as the probing of metabolism in different prostate cancer cells lines (DU145, PC3, and LnCap) [66]. In both studies, the metabolic results were well consistent with those of conventional biochemical investigations, thus showing that the method of hyperpolarization does not affect the metabolic pathways in the investigated cells.

Nevertheless, small amounts of organic solvent (1.7 ± 0.3 mM), alcohol derived from cleavage of the ester (allyl alcohol 1.8 ± 0.4 mM) or metal catalyst (0.7 ± 0.1 µg) are present in the aqueous solution of the HP product.

In vitro cytotoxicity has been investigated [66] and the colorimetric viability assay based on the tetrazolium dye MTT (3-(4,5-dimethylthiazol-2-yl)-2,5-diphenyltetrazolium bromide) showed moderate cytotoxicity (on prostate cancer cells) of the aqueous solution after 24 h treatment. Carrying out the toxicity tests of each single unwanted component of the solution, it was found that the toxic effect is due to the presence of traces of the organic solvent, rather than of the alcohol derived from cleavage of the ester or of the metal catalyst (0.7 ± 0.1 µg).

**Fig. 5:**
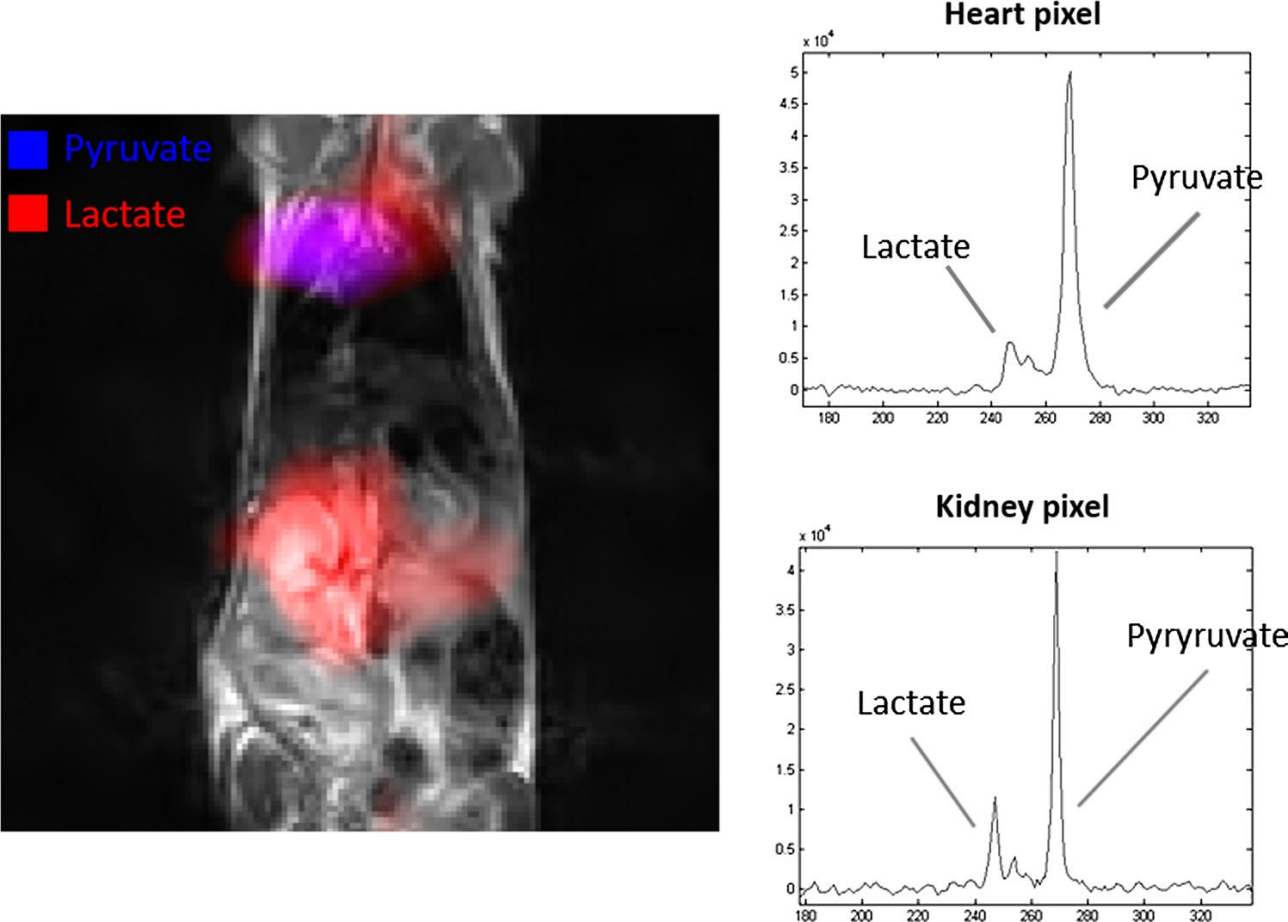
^13^C-chemical shift image reporting pyruvate and lactate distribution acquired on healthy living mouse (BALB/c). The ^13^C-CSI 20 × 20 pixels image was acquired using 1 T MRI (ASPECT 2 M system) 15 s after injection of 170 µl HP [1-^13^C]pyruvate solution (35 Mm). The spatial localization of each metabolite is shown upon overlapping the ^13^C-CSI results to the anatomical ^1^H-image (*T*_2_ weighted image). Each metabolite map is scaled individually and is displayed on blue (pyruvate) and red (lactate) colour scale

An in vivo metabolic study has also been reported on transgenic mice [67] in which the transformation of pyruvate into lactate can be detected in different tissues (Fig. 5).

In conclusion, the reported in-cells and in vivo studies show that the polarization level and the concentration of the metabolites hyperpolarized by means of PHIP-SAH, although seeking for further improvements, are already sufficient for carrying out these metabolic investigations. The impurities still present in the water solution of the HP product do not interfere with metabolism in cells and are compatible with pre-clinical investigations in animal models of diseases, nevertheless advancements are necessary, concerning the purity of the product, in a perspective of in-human applications. All these aspects will be discussed later.

### [1-^13^C]Fumarate

Fumarate (trans-butenedioic acid) is a metabolite that cells convert into malate or succinate. The use of d-DNP hyperpolarized fumarate to report on fumarase activity was the subject of intense scrutiny [68, 69]. That enzyme is responsible for the interconversion of fumarate and malate and is localized exclusively within the cells cytoplasm and mitochondria. Fumarate enters the cells through the sodium dependent dicarboxylate acid transporters and the fumarate-malate metabolic transformation is observed, using the d-DNP hyperpolarized molecule, only in damaged cells, that are characterized by profoundly altered membranes. From this work it was realized that there was a good correlation between the rate of malate formation and the level of cell necrosis. Thus fumarate appears an excellent reporter on therapeutic treatments leading to tumor necrosis, i.e., of conditions in which the intracellular fumarase is allowed to interact with the administered hyperpolarized fumarate.

Besides tumors, the diagnostic potential of fumarate was also assessed in the case of acute kidney injury for its ability to distinguish between acute tubular necrosis (ATN) and glomerular-necrosis (GN) [69]. In fact a significant increase of the [^13^C]malate resonances was observed in the kidneys of mice with ATN (induced by Folic Acid treatment) at the early onset of the disease before histological changes were detectable. No such increase was observed in kidneys with acute GN.

Due to these reasons, parahydrogen hyperpolarization of fumarate is quite appealing for metabolic studies. The vast majority of the parahydrogenation reactions reported in the literature make use of rhodium based complex that leads to cis-hydrogenation of substrates. Using this catalyst, the acetylene dicarboxylate precursor readily forms maleate, i.e., the cis-isomer of the butanedioic acid. Since maleate is a toxic compound, in the context of PHIP applications, only complete hydrogenation to succinate leads to a useful metabolite.

**Fig. 6:**
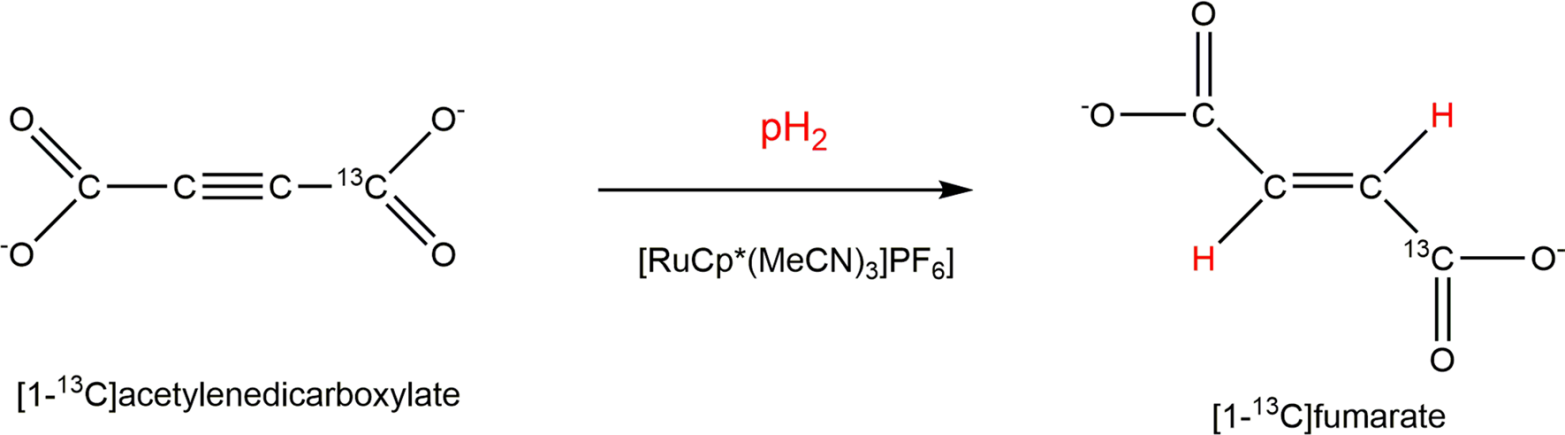
Reaction scheme showing the chemical addition of para-enriched hydrogen to an unsaturated [1-^13^C]acetylene dicarboxylate precursor, to yield [1-^13^C]fumarate

It was shown, recently, that fumarate can be obtained from the pairwise hydrogenation of an acetylene dicarboxylate (ADC) precursor in water [70], using a ruthenium-based catalyst [RuCp*(MeCN)_3_]PF_6_] to achieve the necessary trans-hydrogenation (Fig. 6) [71, 72], with a minor formation of the fully hydrogenated succinate product.

Hydrogenation of ADC to fumarate, using para-hydrogen, does not lead to hyperpolarization, because the two alkenic hydrogen atoms are chemically and magnetically equivalent. This situation changes when the fumarate is ^13^C enriched, either in one (C-1) or in two (C-1 and C-4) positions as the presence of different J_CH_ couplings introduce magnetic asymmetry and consequently loss of the singlet state. Actually, as the two protons remain close to the magnetic equivalence, it was shown that an enhanced ^13^C signal can be obtained by applying a specific pulse sequence such as S2hM (singlet to heteronuclear magnetization) [73]. The reported experimental work-up relied on: (i) hydrogenation of disodium acetylene [1-^13^C]dicarboxylate with parahydrogen in the presence of the ruthenium catalyst at 50 °C in the NMR tube inside the magnet; (ii) application of the S2hM pulse sequence optimized for the J-couplings in fumarate and (iii) the ^13^C acquisition. The applied pulse sequence led only to the enhancement of the central peak of the triplet pattern of the ^13^C carboxylate resonance and ^13^C polarization level was 1%. This work reported that, in degassed conditions, ^1^H–*T*_1_ of fumarate is 22.6 ± 0.3 s, but the ^1^H singlet relaxation time *T*_s_ is longer (46 ± 7 s), since the singlet state is immune to intra-pair ^1^H–^1^H dipolar relaxation. ^13^C relaxation is dominated by the chemical shift anisotropy (CSA) mechanism and the relaxation rate is, therefore, inversely related to the magnetic field strength. A very interesting insight to prolong the lifetime of the hyperpolarized fumarate was suggested via the precipitation of fumarate as solid [74]. In the case of fumarate, this task is easily fulfilled by decreasing the pH as the formed fumaric acid is very poorly soluble in aqueous solutions. In principle this approach may pave the way to innovative routes for storing the hyperpolarization for long times, thanks to the extremely low T_1_ of solids.

**Fig. 7:**
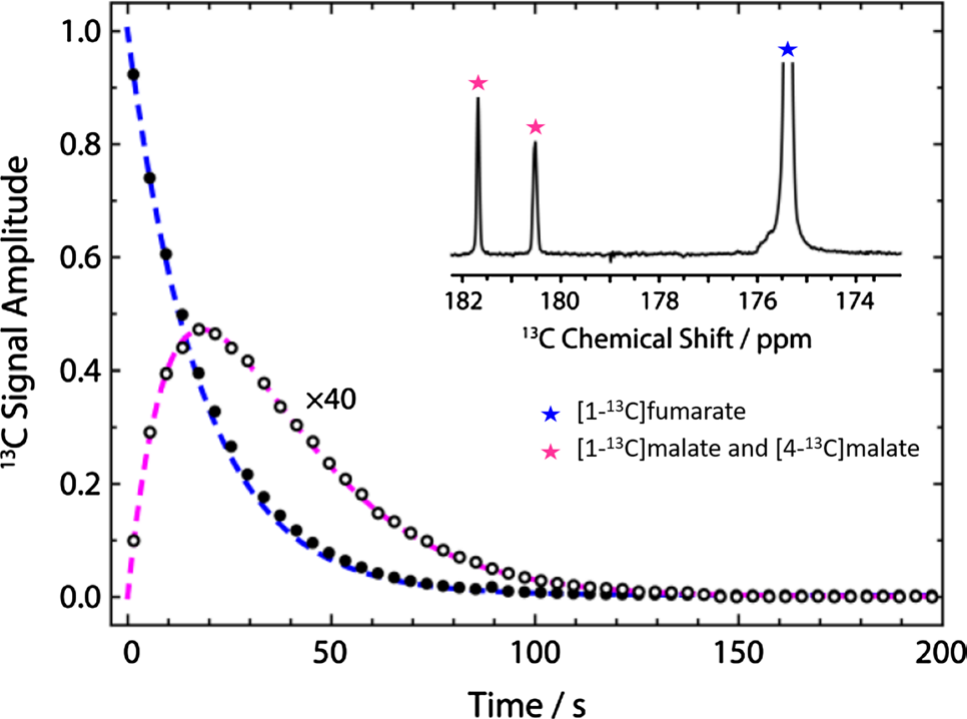
Top: ^13^C carboxylate resonances of fumarate (175.4 ppm) is well distinct from the corresponding resonances of malate (C-1, 181.8 and C-4, 180.6 ppm). Bottom: flux of ^13^C label between fumarate and malate in a suspension of lysed EL-4 tumour cells. The filled black dots represent integrals of [1-^13^C]fumarate and hollow black dots represent integrals of [1-^13^C]malate + [4-^13^C]malate signals

The hyperpolarization of [1-^13^C]fumarate resonance was markedly enhanced using a magnetic field cycle (MFC) [75–77], instead of the rf pulses for spin order transfer (SOT), together with a more efficient hydrogenation method [37]. In fact, highly polarized (24% ^13^C polarization) and concentrated (approx. 45 mM) fumarate solutions were obtained [37] thus showing the relevance of the hydrogenation conditions on the hyperpolarization level attainable using hydrogenative PHIP. The metabolic conversion of PHIP polarized fumarate into malate was reported (Fig. 7), using lysed cells, while it was not observed using intact cells.

### [1-^13^C]Succinate

**Fig. 8:**
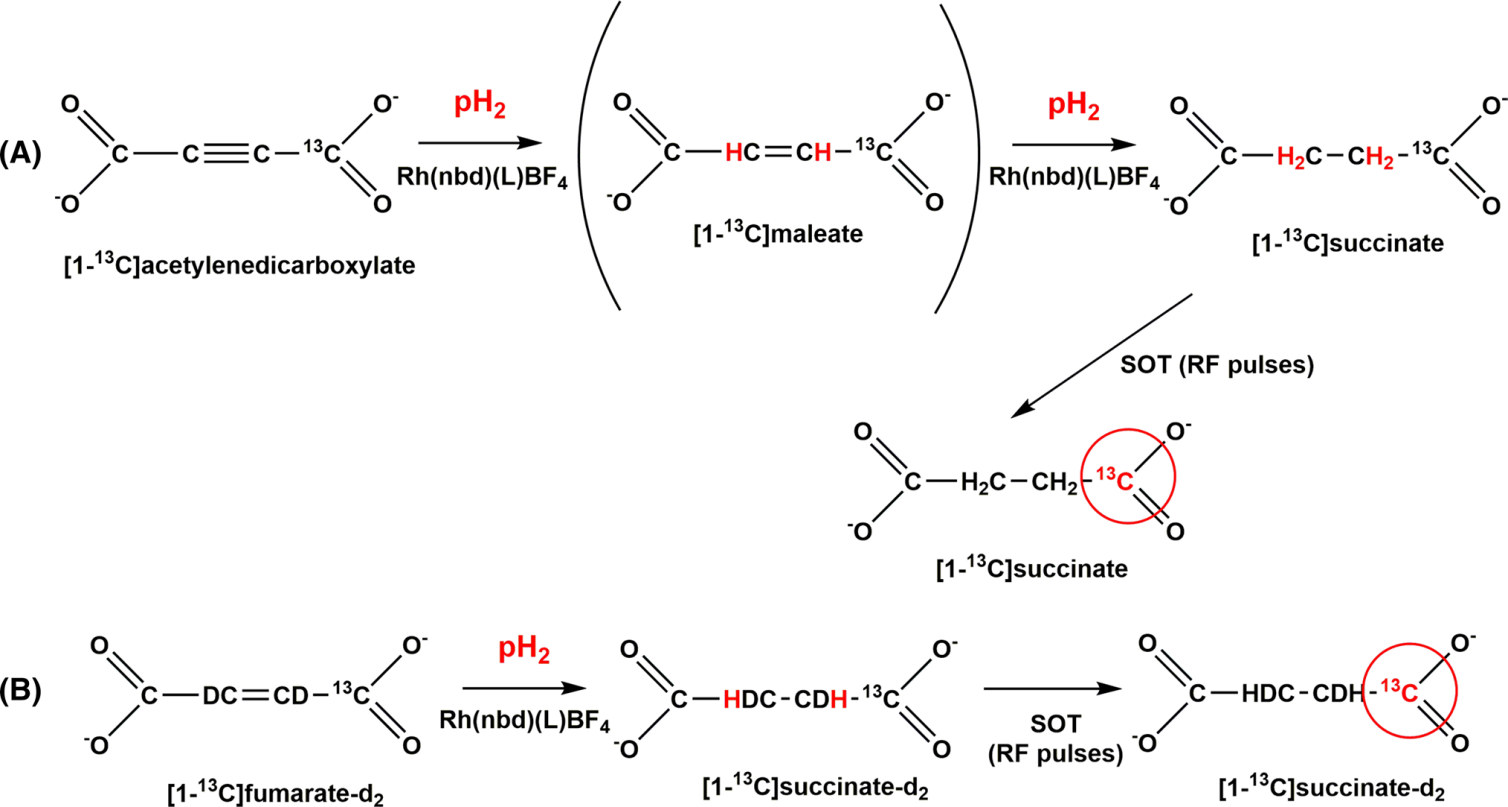
Scheme reporting the key steps of the hyperpolarization procedure: **a** HP-Succinate: hydrogenation of [1-^13^C]acetylenedicarboxylate to [1-^13^C]-maleate and then [1-^13^C]Succinate; **b** HP-Succinate: hydrogenation of [1-^13^C]fumarate-d_2_ to succinate. The reaction are carried out in aqueous solution and are catalysed by the water-soluble Rhodium(I) complex [Rh(nbd)(L)][BF_4_], where *nbd* norbornadiene and *L* is the water soluble ligand 1,4-bis[(phenyl-3-propansulfonate)phosphine]butane. Spin order transfer from parahydrogen to ^13^C is obtained by means of the application of RF pulses

It was recognized relatively quickly that aqueous solutions of hyperpolarized succinate could be obtained directly through the addition of parahydrogen to the unsaturated precursors represented by acetylene dicarboxylate and fumarate [78, 79]. Figure 8 shows the hydrogenation reaction of [1-^13^C]acetylenedicarboxylate in water catalyzed by the water-soluble analog of the most widely applied PHIP catalyst, i.e., a rhodium(I) complex containing a chelating phosphine. The entire hyperpolarization procedure (hydrogenation and heteronuclear hyperpolarization) is conveniently carried out using a PHIP polarizer. Several of these polarizers have been constructed and will be described in another section of this review.

Succinate is involved in the tricarboxylic acid cycle (TCA) cycle, the pivot of cellular metabolism and of the production of energy; numerous diseases, such as cancer or neurological disorders, are correlated to dysfunctions of the TCA cycle. It is evident that being able to analyze these metabolic transformations by means of the hyperpolarization technique is of considerable interest in the diagnostic field. Unfortunately, the metabolic products of succinate cannot be observed using this hyperpolarized substrate maybe due to the slow rate of the transport across the cellular membrane. Nevertheless, it is the first metabolite that has been hyperpolarized by means of hydrogenative PHIP [78] and is still used as a standard compound for polarizer testing [40], tests of RF pulse sequences for spin order transfer [80] and to carry out in vivo imaging [39, 81].

Parahydrogen hyperpolarized [1-^13^C]succinate was obtained through the catalytic parahydrogenation of [1-^13^C]acetylenedicarboxylate [78] in water solution, using a prototype polarizer operating at few mT. The parahydrogenation reaction was carried out in a so-called laminar flow reaction chamber [82, 83] and transfer of parahydrogen spin order to ^13^C magnetization was obtained by means of the application of RF pulses. The HP contrast agent was injected in a rat and a ^13^C MR image was acquired on a 1.5 T MR scanner, using a [1-^13^C]acetate phantom as reference. A significant signal enhancement was observed in the vasculature and head, although with poor spatial resolution.

When [1-^13^C]acetylenedicarboxylate is used as the substrate, toxic [1-^13^C]maleate is obtained as the first hydrogenation product (see Fig. 7), while succinate needs the addition of a second hydrogen molecule. This issue has been addressed using [1-^13^C]fumaric acid-d_2_ as substrate [79] (Fig. 7). The transfer of parahydrogen spin order to ^13^C magnetization was also optimized, considering the dependence of scalar couplings among protons and ^13^C on pH, and 15–20% ^13^C polarization was obtained at the output of the reactor. It must be noticed that, since the transfer of spin order to heteronuclear polarization takes place through J-couplings that depend on pH, ^13^C polarization level can be notably changed by pH. In particular, for pH values close to the pKa of succinic acid, the polarization transfer is hampered by exchange between succinic acid and succinate that disrupts in the ^1^H–^13^C coupling network. Therefore, the pH of the aqueous solution in which the hydrogenation was carried out was set to acidic. Another route to overcome this problem was proposed, where the parahydrogenation of the maleic anhydride led to succinic anhydride [84]. The process was carried out in an organic solvent and the succinic anhydride was then transformed into succinic acid by rapid hydrolysis after the hydrogenation step.

Succinate was also used as a test substrate for automatized PHIP polarizers. [44, 81, 85]. The high ^13^C polarization (25–28%) and a concentration of the substrate (~ 30 mM) achieved using these devices made it suitable for in vivo applications.

The resulting HP [1-^13^C]succinate-d_2_ was used to demonstrate the feasibility of low-field molecular imaging (47.5 mT) in vitro [81] and in vivo[85]. A clear advantage given by low field MRI is the ^13^C T_1_ of [1-^13^C]succinate-d_2_ in aqueous solution, 75 ± 3 s at 47.5 mT instead of 26.6 ± 1.0 s at 4.7 T[79].

The metabolic transformation of PHIP polarized succinate was not observed, as also confirmed using the same metabolite polarized by means of d-DNP. This shows again, that the hyperpolarization method does not interfere with the metabolic processes.

The applicability of this hyperpolarized substrate as angiographic tracer can still be considered a viable possibility, in fact the first in vivo application of a ^13^C hyperpolarized substance, made use of a PHIP polarized probe (hydroxyethyl propionate, HEP) as an angiographic reporter [6].

The use of metabolically inert hyperpolarized substrates as tracers of vascularization and perfusion in tissues was suggested, using d-DNP polarized molecules [86–88] and also water [86, 89]. These agents may be considered useful alternatives to the currently used Gd-based MRI contrast agents that are largely used in the clinical settings to report about organ perfusion and abnormalities in the excretion pathways. Nowadays there is a growing concern about their use, since tiny amounts of gadolinium have been found in the brain and other tissues of patients undergone to multiple administrations of these agents [90]. Although no evidence for a clinical consequence associated with the presence of retained Gd has yet been reported, nevertheless there is an active search for possible metal-free alternatives. As the use of MRI Contrast Agents is a need for the radiologist in a number of diagnostic assessments, the availability of low cost, easily accessible PHIP systems might likely be considered also for this purpose.

### [1-^13^C]phospho-lactate

**Fig. 9:**
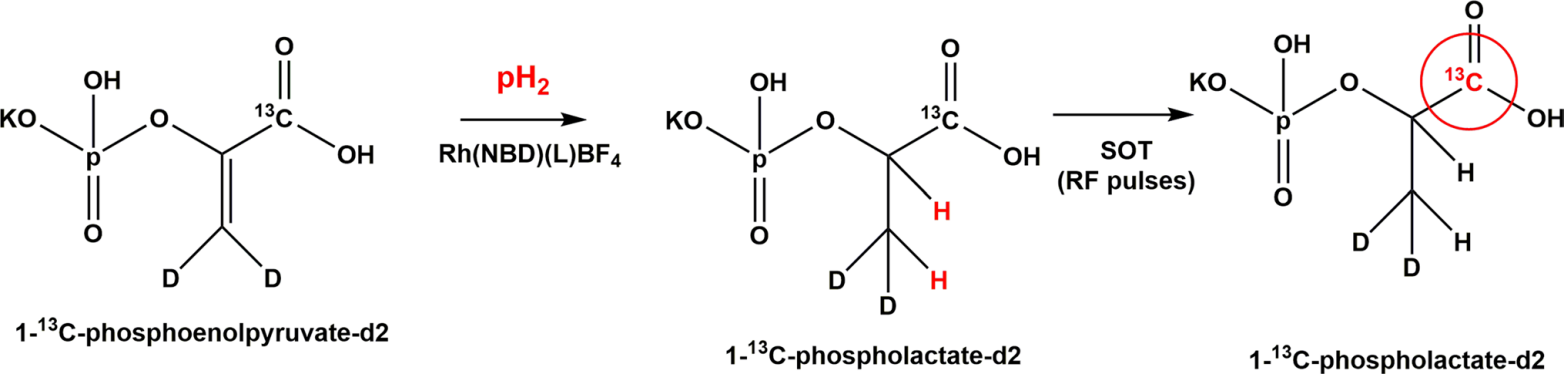
Hyperpolarization of [1-^13^C]phospholactate obtained from parahydrogenation of [1-^13^C]phosphoenoylpyruvate. The hydrogenation conditions are the same as for succinate (Fig. 7). Spin order transfer from parahydrogen to ^13^C is obtained by means of the application of RF pulses

The unsaturated precursor of phospo-lactate (PLac) is the nontoxic [1-^13^C]phosphoenolpyruvate (PEP). Here, the phosphate group stabilizes the double bond adjacent to the oxygen atom and it can be easily enzymatically cleaved without producing toxic byproducts. The hydrogenation catalyst used for the parahydrogenation reaction (Fig. 9) is the same used for parahydrogenation of [1-^13^C]fumarate to [1-^13^C]succinate, i.e., a water soluble rhodium complex, that remains in the aqueous solution of the hyperpolarized product.

A relevant increase of ^13^C hyperpolarization on PLac, with respect to the early reported one for the protonated substrate (1% ^13^C polarization [91]), was obtained using the deuterated derivative [1-^13^C]PEP-d_2_ (15.6 ± 3% ^13^C polarization)[40]. The synthetic route for the deuteration of PLac has been improved recently [92]. Deuteration of this substrate leads to a simplified spin system which is an advantage for the polarization transfer procedure, because hyperpolarization is distributed among three spins instead of five. Being still coupled with the parahydrogen protons on the product molecule, but to a lesser extent than protons, deuterons allow to increase the hyperpolarization level. Selective deuteration of the substrates and, more interestingly, of the catalysts has been employed successfully in non-hydrogenative PHIP (SABRE), in which 50% polarization has been obtained on a derivative of nicotinamide (methyl-4,6-*d*_2_-nicotinate) [21]. Another advantage of selective deuteration is given by the longer T_1_ with respect to the protonated analog, in fact the *T*_1_ of [1-^13^C]PEP-d_2_ is 51 ± 2 s at 5.75 mT, while the T_1_ of the protonated molecule is 36 ± 2 s at the same field. ^13^C polarization was increased also thanks to the optimization of the pH of the water solution in which the hyperpolarization procedure is carried out, in an analogous manner as for succinate hyperpolarization [79].

A good improvement in the concentration of the parahydrogenated product was also achieved, with respect to the initially reported one, yielding ~ 30 mM solution of HP [1-^13^C]PLac-d_2_.

Bio-distribution studies of non-hyperpolarized PLac demonstrated that it undergoes fast dephosphorylation in vivo to yield [1-^13^C]lactate within 1 min.[93]. An in vivo study carried out on mice using hyperpolarized [1-^13^C]-phospholactate 25 mM (∼ 15% ^13^C polarization)[39] showed that the HP PLac can be detected, by means of ^13^C slice-selective gradient echo (GRE) imaging at 4.7 T, in the vasculature, in the heart and in the bladder of the animal. Unfortunately, the spectroscopic differentiation between PLac and lactate was not possible, in vivo, due to small chemical shift difference between the two molecules.

## Open issues and possible solutions

Overall, the in vitro and in vivo studies so far reported for HP molecules generated by h-PHIP clearly demonstrate that they can represent reliable candidates for applications in the biomedical field and, in principle, potential competitors with the hyperpolarized molecules generated by d-DNP. Let’s consider the current weaknesses and possible solutions to make the h-PHIP generated molecules more suitable for the intended applications.

In vivo applications of HP probes need relatively high ^13^C polarization levels and, albeit a few studies have been carried out using 3–4% polarization (see, e.g., [94]), the hyperpolarization level usually reached on d-DNP products is 20–30% [57, 95]. Such values have been reached also for some of the previously reported PHIP substrates [37, 40, 79], but in other molecules, in particular those derived from PHIP-SAH procedure, the attained polarization level is still considerably lower, albeit it is already sufficient to carry out biological investigations. In the second part of this review one of our focus will deal with the determinants of the heteronuclear polarization obtained in PHIP experiments and on the methods that have been developed to tackle this issue.

Next, as the bio-compatibility of the administered solutions is fundamental requisite for in vivo applications; therefore, much attention has to be devoted to reduce as much as possible the presence of the hydrogenation catalyst and organic solvents to avoid toxicity problems. The strategies that have been investigated, to remove the toxic impurities from the aqueous solutions of the hyperpolarized substrates will be reported.

The concentration of the hyperpolarized probes, used for in vivo studies, is rather high, for example, the pyruvate concentration for in-vivo investigations in mice is 80 mM [96] and 230 mM in humans [57]. Since the parahydrogenated product must be obtained in few seconds, to avoid polarization losses during the hydrogenation reaction, the efficiency of the hydrogenation catalyst and of the hydrogenation system must be optimal.

These three points, heteronuclear polarization, biocompatibility of the solution and efficiency of the hydrogenation reaction, will be discussed in the second part of this review.

## From parahydrogen spin order to heteronuclear magnetization

The introduction of parahydrogen induced polarization (PHIP) allowed for dramatically enhanced proton signals, and introduced the challenge of transferring polarization from proton singlet order to heteronuclei characterized by longer *T*_1_. As far as biological applications are concerned, heteronuclear hyperpolarization allows also to benefit from zero background signal.

To get more insight on how heteronuclear hyperpolarization can be observed, and, possibly, increased on parahydrogenated molecules, it is useful to recall some basic quantum–mechanical descriptions of the PHIP phenomenon. A more thorough treatment can be found in other reviews. [14, 97, 98]

The simplest way to get an understanding of how heteronuclei become hyperpolarized, in parahydrogenated molecules, is to consider the population model of the NMR phenomenon. In terms of the spin functions $\alpha$ and $\beta$, the nuclear wavefunctions of the hydrogen molecule are $S_0 = \left. {1 / \sqrt 2} \right| {\alpha \beta - \beta \alpha } \Big >$, $T_{ - 1} = \left| {\alpha \alpha } \right. \Big >$, $T_0 = \left. 1 / \sqrt 2 \right| {\alpha \beta + \beta \alpha } \Big >$, $T_{ + 1} = \big| {\beta \beta } \Big >$, the first is antisymmetric in exchange of the nuclei (this is the *para* state), while the other three are symmetric (the ortho states). Since transitions between states having different symmetry are not allowed, the first state is isolated form the other three and is a singlet state, while the others form a triplet state. At thermal equilibrium, the four states are almost equally populated as only tiny differences stem from the Boltzmann factor that is in the order of 10^–5^. In PHIP experiments one uses hydrogen that is enriched in the parahydrogen isomer, thus the para state is more populated. Different systems have been described that allow to obtain para-enriched hydrogen at different percentage, depending on the temperature reached inside the conversion chamber [99–101].

When hydrogenation, using para-enriched hydrogen, is carried out, the hydrogen molecule undergoes a sudden change and a new spin system is formed. For sake of simplicity we’ll deal with a three spins system (AA’X) in which the third nucleus (X) is a heteroatom and the energy levels are given by the Hamiltonian:

1 $$\rm{\mathcal{H}} = \rm{\mathcal{H}}_Z + \rm{\mathcal{H}}_J ,$$

2 $$\rm{\mathcal{H}}_Z = - \nu_H \left( {I_z^A + I_z^{A^{\prime}} } \right) - \nu_X I_z^X ,$$

3 $$\rm{\mathcal{H}}_J = J_{AA^{\prime}} \left[ {I_z^A I_z^{A^{\prime}} + \frac{{\left( {I_+^A I_-^{A^{\prime}} + I_-^A I_+^{A^{\prime}} } \right)}}{2}} \right] + J_{AX} I_z^A I_z^X + J_{A^{\prime}X} I_z^{A^{\prime}} I_z^X ,$$

where $\rm{\mathcal{H}}_Z$ and $\rm{\mathcal{H}}_J$ are the Zeeman and the J-coupling Hamiltonian, respectively. The nuclear spin wavefunctions can be calculated using the singlet–triplet states for the two protons and the Zeeman states for the heteronucleus as a suitable basis

4 $$S_0 \alpha ,S_0 \beta ,T_{ - 1} \alpha ,T_{ - 1} \beta ,T_0 \alpha ,T_0 \beta ,T_{ + 1} \alpha ,T_{ + 1} \beta .$$

The eigenstates of the new spin system, using this Singlet–Triplet-Zeeman basis, are [101]

$$T_{ - 1} \alpha = \big| {\alpha \alpha \alpha } \Big>$$, $$T_{ - 1} \beta = \big| {\alpha \alpha \beta } \Big>,$$

$$S^{\prime}\alpha = \Big| {c_{11} S_0 \alpha + c_{12} T_0 \alpha} \Big>$$, $$T^{\prime}_0 \alpha = \Big| {c_{21} S_0 \alpha + c_{22} T_0 \alpha} \Big> ,$$

$S^{\prime}\beta = \Big| {d_{11} S_0 \beta + d_{12} T_0 \beta} \Big>$, $T^{\prime}_0 \beta =\Big| {d_{21} S_0 \beta + d_{22} T_0 \beta} \Big> ,$ $T_{ + 1} \alpha = \big| {\beta \beta \alpha}\Big>$, $T_{ + 1} \beta = \big| {\beta \beta \beta} \Big> ,$ where the spin order of parahydrogen is distributed among the states $S^{\prime}\alpha$, $T^{\prime}_0 \alpha$, $S^{\prime}\beta$, and $T^{\prime}_0 \beta$.The states $S^{\prime}\alpha$ and $S^{\prime}\beta$ are more populated, according to the coefficients $c_{11}$ and $d_{11}$ (see [101] for the calculation of these values). As far as hyperpolarization of the heteronucleus is concerned, it can be observed that the transitions between the states $S^{\prime}\alpha$ → $T^{\prime}_0 \beta$ and $S^{\prime}\beta$ → $T^{\prime}_0 \alpha$ are hyper-intense (hyperpolarized), because the population difference between these states is not dictated by the Boltzmann equilibrium, but is related to the para-H_2_ enrichment (see Fig. 9). According to this description, it clearly follows that heteronuclear hyperpolarization can be spontaneously obtained following the parahydrogenation of a substrate, provided that the heteroatom is asymmetrically coupled with the two protons, while the scalar interaction between the two protons is not truncated, i.e., the two protons are strongly coupled. It must be also evidenced that, if the parahydrogenation reaction is carried out at low magnetic field (i.e., in a Altadena experiment) [102] any proton network is strongly coupled and heteronuclear polarization can be observed with any spin system [103].

Heteronuclear hyperpolarization that is spontaneously generated in a parahydrogenation reaction corresponds to longitudinal two spin order and not to net magnetization. Using a product operator description, it can be said that hyperpolarized spin order is obtained, instead of net (longitudinal) magnetization. A short description in terms of product operators will be given too, to discuss the polarization transfer methods that were developed to increase the heteronuclear magnetization.

In terms of product operators, parahydrogen can be written as follows:

5 $$\sigma_{{\text{para}}} = \frac{1}{4} E - \left( {I_z^A I_z^A + I_x^A I_x^A + I_z^A I_x^A + I_y^A I_y^A } \right) = \frac{1}{4} E - \left[ {I_z^A I_z^A + \left( {ZQ_x } \right)^{AA} } \right],$$

where $E$ is the unity matrix, $I_z^A I_z^A$ is the proton-proton longitudinal spin order term and $\left( {ZQ_x } \right)^{AA}$ is the zero quantum coherence term ($\left( {ZQ_x } \right)^{AA} = \left( {I_x^A I_x^A + I_y^A I_y^A } \right))$. Following to the addition of parahydrogen to an unsaturated substrate and the formation of an AA’X spin system, the Hamiltonian changes suddenly. The time evolution of the density operator is given by the commutator of the Liouville-Von Neumann equation:

$$\frac{{d\sigma_{{\text{para}}} }}{dt} = - i\left[ {\sigma_{{\text{para}}} ,\rm{\mathcal{H}}} \right],$$

where $\rm{\mathcal{H}}$ is the Hamiltonian of the AA’X system (1). The two spins order term $I_z^A I_z^A$ commutates with the Hamiltonian, i.e., it remains invariant under the action of the Hamiltonian, and the evolution of the initial parahydrogen polarization can be reduced to the evolution of the zero-quantum coherence term $\left( {ZQ_x } \right)^{AA}$. Starting from this term, we obtain [104]:

$$\left\{ {2\left( {I_y^A I_x^{A^{\prime}} - I_x^A I_y^{A^{\prime}} } \right)I_z^X } \right\} = \left\{ {2\left( {ZQ_y } \right)^{AA} I_z^X } \right\},$$

and proton-carbon spin order $\left\{ {\left( {I_z^A - I_z^{A^{\prime}} } \right)I_z^X } \right\}$.

The (time dependent) density matrix becomes

6 $$\sigma \left( t \right) = \frac{1}{4}E - I_z^A I_z^{A^{\prime}} - a\left( t \right)\left( {ZQ_x } \right)^{AA^{\prime}} - b\left( t \right)2\left( {ZQ_y } \right)^{AA^{\prime}} I_z^X - c\left( t \right)\left( {I_z^A - I_z^{A^{\prime}} } \right)I_z^X .$$

having time-dependent coefficients $a\left( t \right), b\left( t \right)$ and $c\left( t \right)$.

Since various molecules are hydrogenated at different times, the density matrix starts to evolve at different timepoints and, at the end of the hydrogenation, an average density matrix is obtained. The averaged coefficients, calculated by Natterer et al. [104], are.

7 $$\overline{a}\left( t \right) = \left( {\frac{{J_{AA^{\prime} } }}{{J^{\prime} }}} \right),\quad \overline{b}\left( t \right) = 0\quad {\text{and}}\quad \overline{c}\left( t \right) = \frac{{J_{AA^{\prime} } J_{\Delta } }}{{\left( {J^{\prime} } \right)^2 }}\overline{c}\left( t \right) = \frac{{J_{AA^{\prime} } J_{\Delta } }}{{\left( {J^{\prime} } \right)^2 }},$$

in which $J^{\prime} = \sqrt {J_{AA^{\prime}}^2 + \left( {J_{\Delta } } \right)^2 }$ and $J_{\Delta } = \frac{{J_{AX} - J_{A^{\prime}X} }}{2}$.

Therefore, it results clearly that the term $\left( {ZQ_y } \right)^{AA^{\prime}} I_z^X$ is averaged to zero and heteronuclear hyperpolarized signals are given by the spin order term $\left( {I_z^A - I_z^{A^{\prime}} } \right)I_z^X$, that corresponds to antiphase ^13^C signals, as shown using the spin states populations.

Several different methods have been developed to optimize the transfer of parahydrogen spin order to longitudinal heteronuclear magnetization, some based on magnetic field cycling and other that make use of RF pulse sequences.

### Magnetic field cycle

**Fig. 10:**
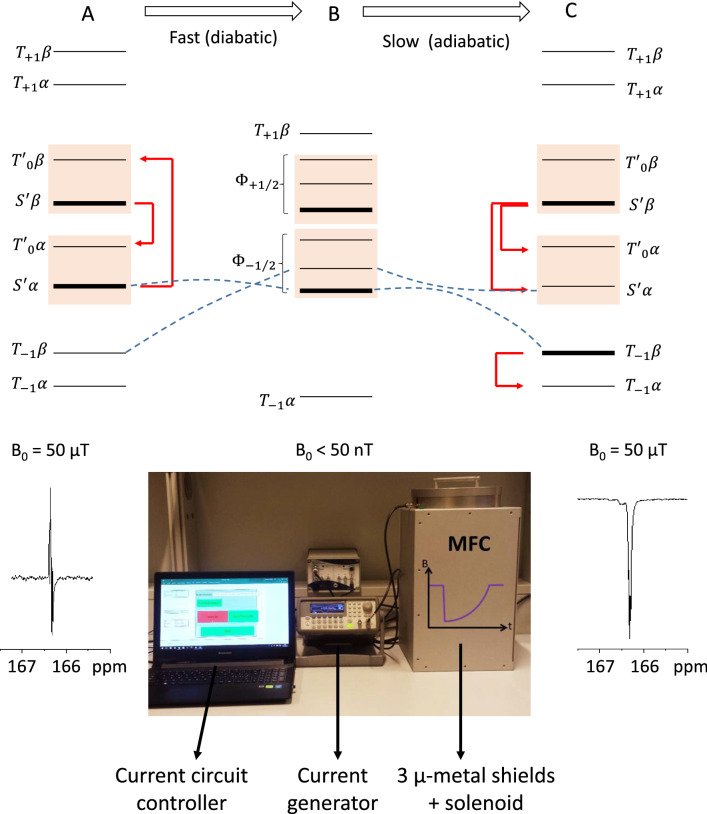
Upper panel: diagram representing the population of the nuclear spin states in a AA’X system: **a** population of the nuclear spin state after the parahydrogenation reaction at geomagnetic field (~ 50 µT); **b** after a diabatic (fast) decrease of the magnetic field to few nT, the population of the spin stats are re-arranged; **c** when the magnetic field is adiabatically increased again. Dashed lines evidence the crossing (on the left) and avoided crossing (LAC, on the right) of the spin states in the two asymmetric passages. The red arrows represent the hyper-intense (hyperpolarized) transitions of the heteronucleus. Lower panel: ^13^C-NMR signal of a symmetric product: **a** before the application of MFC, antiphase hyperpolarized signals are observed for the heteronucleus, **c** after the application of magnetic field cycle, net magnetization is obtained. In the central panel is reported a photo of the system used for the application of MFC: it consists in a mu-metal shield, an arbitrary wavefunction generator for the application of controlled current to the coil and a computer to control the applied current. More details can be found in [111]

Longitudinal polarization on a heteronucleus has been obtained, for the first time, following a diabatic-adiabatic magnetic field cycling (MFC) process. [5, 75] In this process, the hydrogenation is performed in a static magnetic field high enough to warrant the truncation of all scalar interaction between different nuclear species. Then the field is suddenly (diabatically) decreased so that the populations of the spin states are redistributed, with respect to high field. When the field is slowly (adiabatically) raised to the initial value, the populations of the instantaneous eigenstates remain constant. The populations of the original eigenstates are rearranged and the systems displays a heteronuclear NMR spectrum, where the transitions are predominantly in-phase (Fig. 10).

For fields greater than the geomagnetic field (~ 50 $\mu {\text{T}}$) the high field regime is fulfilled, i.e., the scalar interaction between protons and carbon is truncated, and the effective Hamiltonian is, for the AA’X system, the same as in Eq. 1.

Immediately after the hydrogenation step, the magnetic field is suddenly dropped to few tens of nano Tesla (30–50 nT), where the J-coupling Hamiltonian differs from the high-field one (Eq. 3) by the addition of flip-flop terms ($\frac{1}{2}\left( {I_+^A I_-^X + I_-^A I_+^X } \right)$ and $\frac{1}{2}\left( {I_+^{A^{\prime}} I_-^X + I_-^{A^{\prime}} I_+^X } \right)$) between heteronuclear spin pairs:

8 $$\rm{\mathcal{H}}_J = J_{AA^{\prime}} \left[ {I_z^A I_z^{A^{\prime}} + \frac{{\left( {I_+^A I_-^{A^{\prime}} + I_-^A I_+^{A^{\prime}} } \right)}}{2}} \right] + J_{AX} \left[ {I_z^A I_z^X + \frac{{\left( {I_+^A I_-^X + I_-^A I_+^X } \right)}}{2}} \right] + J_{A^{\prime}X} \left[ {I_z^{A^{\prime}} I_z^X + \frac{{\left( {I_+^{A^{\prime}} I_-^X + I_-^{A^{\prime}} I_+^X } \right)}}{2}} \right].$$

The eigenstates can be calculated, using the basis (4):

$${\Phi }_{ - 3/2} = T_{ - 1} \alpha \quad {\Phi }_{ + 3/2} = T_{ + 1} \beta ,$$

$${\Phi }_{ - 1/2} = c_{11} { }T_{ - 1} \beta { } + c_{12} S_0 \alpha + c_{13} T_0 \alpha \quad {\Phi }_{ + 1/2} = d_{11} { }T_{ + 1} \alpha { } + d_{12} S_0 \beta + d_{13} T_0 \beta ,$$

$$\Phi^{\prime}_{ - 1/2} = c_{21} T_{ - 1} \beta + c_{22} S_0 \alpha + c_{23} T_0 \alpha \quad \Phi^{\prime}_{ + 1/2} = d_{21} T_{ + 1} \alpha + d_{22} S_0 \beta + d_{23} T_0 \beta ,$$

$$\Phi \prime \prime_{ - 1/2} = c_{31} T_{ - 1} \beta + c_{32} S_0 \alpha + c_{33} T_0 \alpha \quad \Phi \prime \prime_{ + 1/2} = d_{31} T_{ + 1} \alpha + d_{32} S_0 \beta + d_{33} T_0 \beta ,$$

where the three states $(T_{ - 1} \beta - S_0 \alpha - T_0 \alpha )$ and $(T_{ + 1} \alpha - S_0 \beta - T_0 \beta )$ are mixed. The population of the states at zero field depends, again, on the coefficients $c$ and $d$ of the $S_0 \alpha$ or $S_0 \beta$ basis states.

During the adiabatic transfer from low field to intermediate field, the Eigenstates at zero field evolve slowly into the Eigenstates characteristic of an intermediate field and the populations of the instantaneous Hamiltonian are left unchanged. The Eigenstates and eigenvalues for an arbitrary field can be calculated using numerical methods and the continuous transformation from low to intermediate field can be monitored, to find the correlations between zero and intermediate field. The result is a net polarization of the ^13^C spins instead of the initial ^1^H-^13^C spin order. The theoretical efficiency of spin order transfer, i.e., the maximum polarization obtainable, depends on the J-couplings and on the number of spins involved. It is higher when the system is formed by only three spins (the two protons from parahydrogen and the heteroatom) and becomes lower when the number of spins increases.

An alternative field-cycling method has been recently proposed, together with an accurate theoretical description of the dynamics of the spin system, in which the applied magnetic field is adiabatically swept from a negative to a positive value, passing through zero. [77]

Another strategy to achieve spin mixing is by means of strong resonant field, that is, under spin-locking conditions. The application of resonant RF excitation at the NMR frequencies of protons and heteronuclei brings the system to a level anti-crossing (LAC, also termed avoided level crossing), where efficient spin mixing occurs [105]. This sequence employs two channels (^1^H and heteronuclear) at the same time and provides a route to controlled and selective isotropic mixing between different nuclei.

### RF pulses based methods for spin order transfer

Several pulse sequences have been developed to transform the singlet order of parahydrogen into heteronuclear magnetization.

The first NMR pulse sequences to transfer the parahydrogen induced polarization to heteronuclei were reported by Haake et al. [106] and are based on the INEPT sequence. They were named PH-INEPT and PH-INEPT+, and were applied to molecules in which heteronuclear polarization was not spontaneously created. This occurs when the two parahydrogen protons are added to chemically different sites, in a strong magnetic field, and the weak coupling condition among protons applies. If a three spins system, formed by the two parahydrogen protons ($$H_A$$ and $$H_X$$) and one heteroatom ($$X)$$, is considered, the Zeeman ($$\rm{\mathcal{H}}_Z )$$ and J-coupling Hamiltonian ($$\rm{\mathcal{H}}_J )$$ (Eq. 1) become, in this case

9a $$\rm{\mathcal{H}}_Z = - \nu_{H_A } \left( {I_z^{H_A } } \right) - \nu_{H_X } \left( {I_z^{H_X } } \right) - \nu_X I_z^X ,$$

9b $$\rm{\mathcal{H}}_J = J_{H_A H_X } \left( {I_z^{H_A } I_z^{H_X } } \right) + J_{H_A X} \left( {I_z^{H_A } I_z^X } \right) + J_{H_X X} \left( {I_z^{H_X } I_z^X } \right).$$

These are the so-called Pasadena experiments [12]. The spin order term $I_z^{H_A } I_z^{H_X }$ commutes with the Hamiltonian; therefore, it remains invariant, while from the evolution of the zero-quantum coherence ($ZQ_x = \left( {I_x I_x + I_y I_y } \right)$), the term $2\left( {I_y^{H_A } I_x^{H_X } - I_x^{H_A } I_y^{H_X } } \right)I_z^X$) is formed. Both zero quantum coherence terms are time dependent and oscillate around zero, so they are zero-averaged and the parahydrogen derived density operator simplifies to $\sigma_{{\text{Pasadena}}} = \frac{1}{4}E - I_z^{H_A } I_z^{H_X }$. The INEPT based RF pulse sequences, such as the PH-INEPT, PHINEPT+, l-PHINEPT+ [80] and ESOTHERIC [35] act on this part of the parahydrogen density operator.

Other pulse sequences were developed to improve the efficiency of spin order transfer from parahydrogen to heteronuclei. The sequence developed by Golman and co-workers [76, 107] exploits the term $\left( {ZQ_y } \right)^{AA^{\prime}} I_z^X$ (that results according to Eq. 6 for an AA’X spin system). When using this sequence, the hydrogenation step takes place under a strong RF irradiation at the proton Larmor frequency. This disconnects the singlet state from the triplet state, thereby maintaining it until the hydrogenation ends. At this point it is stopped and all the spins starts to evolve coherently. The first part of the sequence tends to maximize the term $\left( {ZQ_y } \right)^{AA^{\prime}} I_z^X$, while the second part converts this term into ^13^C net polarization.

The efficiency of the pulse sequence in transferring spin order to ^13^C magnetization depends on the relative strengths of the three internuclear J-couplings. In particular, it is close to unity if the so-called Goldman angle $\theta = arctan\frac{{J_{HH} }}{{\frac{1}{2}\left( {J_{H1C} - J_{H2C} } \right)}}$ [76] is near to 45°, i.e., for molecules in which parahydrogen protons are coupled with the target heteronucleus through relatively large scalar couplings. If this is not the case, the work by Kadlecek et al. proposed a more general solution [108].

Other sequences for spin order transfer have been initially developed to store hyperpolarized magnetization into singlet order. In fact, it may be noticed that hydrogenation reactions with para-enriched hydrogen yield a product that has strongly enhanced nuclear singlet order; therefore, these techniques can also be exploited to convert the parahydrogen derived singlet order into magnetization. The SLIC [109] and the S2hM (Singlet to heteronuclear Magnetization) sequence [26, 73, 110] were adapted to perform singlet to heteronuclear magnetization transfer. These sequences operate in the near-equivalent regime, in which the two nuclei involved in the singlet state have a much stronger J-coupling between them than any other interaction that breaks the symmetry, such as chemical shift difference or asymmetric J-couplings with other nuclei. The application of these pulse sequences involves manipulations at low magnetic field, to minimize proton chemical shift differences. Problems may arise, since low-field electromagnets suffer from instability and drift and have a limited homogeneity. The robustness of these sequences to resonance offsets has also been investigated [110].

## Hydrogenation catalysis

In PHIP procedures, the hydrogenation step is crucial, because it is instrumental to transform the singlet order of parahydrogen into hyperpolarization. This process takes place provided that the two protons of hydrogen are pairwise added to the same substrate molecule.

It is important to consider that the proton polarization, in the product molecules, is invariantly limited by the catalytic process. Thus the proton polarization observed in a hydrogenated substrate is substantially lower than the ideal case, in which the singlet order is completely transferred to the product molecule. This is usually observed when Pasadena or Altadena experiments are carried out (see, e.g., [63, 111]) and can be termed the polarization transfer efficiency (PTE) [112]. PTE is the ratio between the polarization experimentally obtained on the product (regardless of the T_1_ relaxation of the hyperpolarized product) and the ideal case in which no polarization is lost during the catalytic cycle.

If the proton singlet order would be transferred directly to the product, without being affected by the reaction intermediates, PTE would be 1, but, in real cases, the hyperpolarization observed on the products depends on decoherence in the hydrogenation intermediates, where singlet and triplet states are mixed. This was already evident in the early studies about PHIP [104], and then nicely described by Berner et al.[44]. It was observed that hydrogenation intermediates strongly affect resulting pattern of hyperpolarized NMR signals. Therefore, the hydrogenation catalysts, the stability of reaction intermediates and, more generally, the conditions of the hydrogenation reaction (hydrogen pressure, temperature, solvents) are crucial in determining the polarization level on protons and, consequently, on heteronuclei [113].

The most widely applied hydrogenation catalyst for PHIP hyperpolarization is a rhodium complex containing a bi-dentated phosphine. The hydrogenation pathway carried out by this kind of complexes, the so-called unsaturated route, is such that, firstly, the unsaturated substrate is added to the metal center and then the hydrogen molecule is almost directly transferred to the coordinated substrate, with very short-living, and undetectable, reaction intermediates [114, 115]. Thanks to this pathway, mixing between the singlet and the triplet states should be minimized on intermediates, nevertheless the PTE is still far from unity. In a recent publication, the effect of different ligands on hydrogenation and hyperpolarization efficiency [116] has been explored and it has been shown that rhodium complexes with electron-donating bisphosphine ligands appear to be more effective than conventional rhodium catalysts.

A fundamental requirement for the application of PHIP polarized molecules to biological investigations is that the solution of the hyperpolarized probe molecule must be aqueous, therefore, organic solvents (such as methanol-d_4_ and acetone-d_6_), in which these hydrogenation catalysts are usually employed, cannot be used. This is a strong limitation, because hydrogenation catalysis is more efficient in organic solvents than in water, thanks also to higher H_2_ solubility. The hydrogenation and phase extraction method has been introduced for parahydrogen hyperpolarization of lipophilic substrates such as anhydrides or esters that can be transformed into hydrophilic molecules by means of hydration or hydrolysis reactions [32, 84]. In this case, the hydrogenation reaction is carried out in a hydrophobic organic solvent that, following to the addition of the aqueous solution, forms a separate phase in which the hydrogenation catalyst is retained. The advantages are given by the fact that hydrogenation is more efficient thanks to the use of the organic solvent and, at the same time, the catalyst is not present in the aqueous solution of the final product. However, it can be applied only to a limited number of substrates.

A water-soluble bis-phosphine ligand has also been widely applied to obtain the analogous rhodium complex suitable for hydrogenation in aqueous phase [79, 82, 91], nevertheless, in this case, a limitation to in vivo applications might be given by the concentration of the rhodium complex in the solution of the hyperpolarized product. Early investigations of rhodium based hydrogenation catalyst have shown relatively low toxicity [117], anyway more detailed studies have to be planned. The use of metal scavengers has been proposed, for removal of the Ir catalysts used in SABRE hyperpolarization [118] and the same principle, using an appropriate scavenger, could be applied for removal of the rhodium complexes.

Precipitation and filtration of the hyperpolarized products is an intriguing method for the purification of the hyperpolarized molecules. The preliminary studies carried out using thermally polarized fumarate [119] demonstrate that this molecule, thanks to its specific physic-chemical properties, can be easily precipitated from an aqueous solution at acidic pH and then readily dissolved at neutral pH. To minimize relaxation, it is important to maintain the sample at high magnetic field during the passage between different states (liquid to solid and vice-versa).

Heterogeneous catalysts are attractive, because they can be separated from the solution of the product by filtration, but, unlike homogeneous hydrogenation which takes place on a well-defined metal center, heterogeneous hydrogenation proceeds over a surface of a metal cluster, where dissociative chemisorption of hydrogen molecules prevents pairwise addition of parahydrogen and leads to loss of singlet spin order. The use of immobilized complexes seemed the most straightforward way to combine the advantages of homogeneous and heterogeneous catalysis, and heterogeneous PHIP has been observed in the liquid phase using rhodium complexes immobilized over SiO_2_ [120]. Rather unexpectedly, supported metal catalysts leaded to PHIP effect that were observed in the gas phase (Pt/Al_2_O_3_ and Pd/Al_2_O_3_ [121]) and in solution (Rh/TiO_2_ and Rh/AlO(OH) [122–124]). The rhodium particles supported on beads of TiO_2_ (2–3 mm) were also applied to the aqueous phase hydrogenation of vinyl acetate [125, 126]. Unfortunately, the low pairwise selectivity of hydrogenation limited the hyperpolarization level. Surface diffusion can be limited by ligands that binds to the catalyst surface and stable, water soluble nanoparticles capped with amino-acid ligands (l-cysteine and *N*-acetylcysteine) have been tested for parahydrogenation of different substrates [45, 127]. Nevertheless, the concentration of substrates used with these catalysts are usually quite small (less than 1 mM) and the filtration procedure to separate 2 nm nanoparticles appears quite challenging and cannot be mitigated by decantation of the HP fluid during a time frame that is compatible with the ^13^C T_1_.

PtSn intermetallic nanoparticles, encapsulated in mesoporous silica (PtSn@mSiO_2_ [128]) were also reported to give intense polarized ^1^H-NMR signals in D_2_O, thanks to the effect of incorporated Sn on the pairwise selectivity of hydrogenation.

In general, all these strategies need to be optimized and investigated to reach the final goal and to obtain a pure aqueous solution of the h-PHIP polarized substrate, suitable for in-human investigations.

## PHIP polarizers

Since the first in vivo application of PHIP polarized substrates [5], which was also the first ^13^C MRI in-vivo, several automated systems have been developed for the production of doses of hyperpolarized molecules that can be suitable for imaging purposes.

In the so-called laminar flow polarizers, hydrogenation occurs in a reaction chamber pressurized with parahydrogen (7–8 bar), in which the solution of reagents (substrate and catalyst) is injected. The hydrogenation chamber is placed in a low filed NMR unit made of low electromagnetic field (1.4 mT in the first reported set-up [83] and ~ 5mT in other developed successively [39]) equipped with coils for the application of the RF pulse sequences that allow the transfer of spin order from parahydrogen protons to ^13^C magnetization [84, 129, 130]. Thanks to the fact that the fast parahydrogenation reaction is carried out in a low-field MR unit, it can be synchronized with the pulse sequence. During the reaction time course, continuous wave decoupling can be applied to freeze the singlet state, a requirement that arises, since various molecules are hydrogenated at different times and the density matrix would, therefore, start to evolve at different time points, so spin order partly is lost. In a particular set-up, placing the hydrogenation chamber in a 48 mT Halbach magnet, allows the in-situ detection of parahydrogen hyperpolarization [131].

Unfortunately, none of these systems is commercial, but detailed descriptions are provided by the literature. An automated PHIP-polarizer equipped with relatively low cost open source software and hardware which allows replication has been reported. The reactor is settled at *B*_0_ field of ∼ 5.75 mT; the hydrogenation can be performed in the range 40–75 °C and up to 21 atm (usually ∼ 6 atm of 90% parahydrogen gas are used); during the reaction, ^1^H decoupling is provided by the RF pulse sequence. In situ ^13^C polarimetry of the produced hyperpolarized contrast agent can also be achieved.

An alternative way to overcome the necessity of a polarizer has been introduced by the synthesis amid the magnet bore, a dramatically enhanced nuclear alignment (SAMBADENA) [44, 113, 132]. The production of HP tracers can be achieved by hydrogenating the precursors in a reactor that is placed inside the bore of a commercial MRI system. In this case, the hardware of the system itself is used for the application of rf pulses for spin order transfer and for the detection of the MR signal, at the same site of polarization.

Another kind of set-up is often used, in PHIP experiments, in which the reaction is carried out by means of parahydrogen bubbling, at high pressure, in a NMR tube containing the solution of substrate and catalyst. In this case, the reaction can take place into the NMR spectrometer and the control of parahydrogen pressure and flow can be interfaced with the spectrometer, via magnetic valves [133], thus allowing to coordinate the reaction and the RF pulses. This kind of set-ups is easier to build than the spray-injection polarizers operating at high pressure and temperature. Unfortunately, the hydrogenation efficiency is considerably lower and the substrate conversion takes several seconds, while the ultra-fast reaction (3–5 s) that can be obtained in spray-injection polarizers prevents ^1^H hyperpolarization losses due to relaxation during the time course of the reaction.

## Conclusions and outlook

The access to hyperpolarized bio-molecules that act as in vivo reporters has paved the way to study metabolism with Magnetic Resonance Imaging and Spectroscopy. Whereas most of currently reported in in vivo studies have been carried out with molecules hyperpolarized by the d-DNP technology, there is sufficient evidence to anticipate that also PHIP generated systems will have a role in this field. Whereas, in d-DNP the source for the polarization transfer is provided by unpaired electrons, in the case of PHIP the source is represented by parahydrogen. As outlined in the above paragraphs, a limitation of PHIP deals with the need of the availability of an unsaturated precursor for the target molecule. Hydrogenative PHIP has found some solution to this fundamental issue, nevertheless some weaknesses can still be noticed, that are the presence of toxic impurities and, in some cases, the hyperpolarization level.

Concerning the toxic impurities in the solutions of the HP probes, it must be evidenced that, in some cases, these have been reduced to little more than traces [40, 66]. Furthermore, the metabolic studies carried out, with some of the reported substrates, in-cells [65, 66] and in-vivo [67, 117] showed that the HP solutions are well tolerated and the results are consistent with biological investigations. This provides evidence that PHIP polarized substrates are promising probes for in vivo biological investigations. Nevertheless, work has still to be done to reduce those substances, especially on a clinical translation perspective.

As far as the increase of the hyperpolarization level is concerned, h-PHIP can already benefit from the research which has been dedicated to improve the spin order transfer from parahydrogen protons to heteronuclei, in different kind of products. Besides these quantum–mechanical aspects, much attention has still to be focused on the chemistry of the hyperpolarization procedure, in particular on the hydrogenation catalysis. Nevertheless, the advancements in this field must always keep an eye on the biocompatibility of the final solution, as well as on the high amount of the HP product that must be obtained, in few seconds.

In the end, the development of a dedicated polarizer, able to provide highly standardized HP products, endowed all the requirements (bio-compatibility of the solutions, concentration of the HP gent and hyperpolarization level) needed for in-vivo pre-clinical studies, in different research sites, will be necessary to foster the clinical translation of these promising HP probes.
